# Oxaliplatin-Based Adjuvant Chemotherapy without Radiotherapy Can Improve the Survival of Locally-Advanced Rectal Cancer

**DOI:** 10.1371/journal.pone.0107872

**Published:** 2014-09-22

**Authors:** Jun Li, Yue Liu, Jian-Wei Wang, Yang Gao, Ye-Ting Hu, Jin-Jie He, Xiu-Yan Yu, Han-Guang Hu, Ying Yuan, Su-Zhan Zhang, Ke-Feng Ding

**Affiliations:** 1 Department of Surgical Oncology, Second Affiliated Hospital, Zhejiang University School of Medicine, Hangzhou, Zhejiang Province, China; 2 The Key Laboratory of Cancer Prevention and Intervention, China National Ministry of Education, Hangzhou, Zhejiang Province, China; 3 Department of Medical Oncology, Second Affiliated Hospital, Zhejiang University School of Medicine, Hangzhou, Zhejiang Province, China; Harvard Medical School, United States of America

## Abstract

**Objective:**

To assess the impact of oxaliplatin-containing adjuvant chemotherapy on the survival of patients with locally-advanced rectal cancer.

**Methods:**

Data on patients with pathologically-confirmed T3/4 or N1/2 rectal cancer who accepted radical surgery at our center from January 2002 to June 2009 were reviewed retrospectively. The patients' 5-year overall survival (OS), disease-specific survival (DSS), and recurrence-free survival (RFS) were analyzed by comparing those who accepted radical surgery only (Group S) with those who accepted radical surgery and oxaliplatin-containing adjuvant chemotherapy (Group SO).

**Results:**

A total of 236 patients were analyzed (Group S 135; Group SO 101). Group S patients were older and had a higher proportion with stage II disease and more perioperative complications than those in Group SO (*P*<0.05). The OS and DSS of patients with stage III disease under 50 years of age or with mucinous adenocarcinoma were higher in Group SO than Group S (*P*<0.05). In addition, the OS of patients with stage N2b disease was higher in Group SO than Group S (*P* = 0.016), and the OS of patients with stage N1a or N2b disease who received more than 8 weeks of oxaliplatin-containing chemotherapy was also higher in Group SO than Group S (*P*<0.05). Although the OS and DSS of patients with stage II disease in Group SO showed a tendency towards improvement, the differences between the groups were not statistically significant.

**Conclusion:**

Adjuvant oxaliplatin-containing chemotherapy can improve the survival of patients with locally-advanced low and middle rectal cancers in comparison with observation. Randomized, prospective trials are warranted to confirm this benefit of oxaliplatin for rectal cancer.

## Introduction

There are obvious differences in adjuvant therapy schedules between colon cancer and rectal cancer. Chemotherapy regimens containing oxaliplatin, such as FOLFOX and XELOX, have been the standard adjuvant therapy for advanced colon cancer for some time [1]–[3]. For locally-advanced rectal cancer, preoperative 5-fluorouracil (5-FU) or capecitabine with radiotherapy is recognized as the standard therapy because of the decreased local recurrence rate and improved survival [4]–[6]. The postoperative use of oxaliplatin in rectal cancer resulted from an extrapolation of the available data in colon cancer. However, 2 phase III trials in patients with locally-advanced rectal cancer reported that the addition of oxaliplatin to the standard of 5-FU or capecitabine and radiation did not result in an increased tumor response but did cause greater toxicity [7], [8]. Currently, no trials have assessed the effect of single adjuvant chemotherapy regimens containing oxaliplatin for rectal cancer as the key role of radiotherapy was established 2 decades ago.

The first National Guideline for the Diagnosis and Treatment of Colorectal Cancer in China, which referred to the US National Comprehensive Cancer Network (NCCN) guideline, was published in 2010 [9]. Prior to 2010, the treatment scheme for locally-advanced low and middle rectal cancers in China was different to that in western countries [10]. Most surgeons in China accepted the concept of total mesorectal excision (TME) from the beginning of the 21st century. However, the role of radiotherapy had been underrated for some time. Consequently, Chinese surgeons preferred to perform surgery primarily and advised patients with T3/4 disease or with regional lymph node involvement to receive intensive postoperative combination chemotherapy containing oxaliplatin and 5-FU or capecitabine secondarily. Radiotherapy was used only for rectal cancer patients with unresectable tumors or uncertain resection margins. Obviously, this treatment scheme was similar to that used for colon cancer in western countries, even though there was a lack of supporting evidence from clinical trials. Although the scheme was subsequently abandoned, it provided the opportunity to study the effect of single adjuvant chemotherapy containing oxaliplatin without radiation for patients with rectal cancer.

In this retrospective study, we reviewed the available data on patients with locally-advanced rectal cancer who underwent surgery at our hospital prior to 2010. The efficacy of adjuvant chemotherapy containing oxaliplatin was analyzed by comparing survival differences between patients who had received surgery only (Group S) and those who had received surgery and adjuvant chemotherapy that included oxaliplatin (Group SO).

## Patients and Methods

### Patients

The colorectal cancer follow-up database at the Zhejiang University Cancer Institute (formerly the Key Laboratory of Cancer Prevention and Intervention, China National Ministry of Education) was reviewed. The follow-up deadline was April 1, 2013. Patients who underwent radical rectal cancer surgery from January 2002 to June 2009 were eligible for analysis. Inclusion criteria included: adenocarcinoma or mucinous adenocarcinoma ≤12 cm from the anal verge; pathologically-confirmed T3/4 or N1/2 disease in patients without a prior history of malignancy; neither transanal nor trans-sacral resection; single tumors or multiple tumors resectable by one operation; absence of distant metastases; and planned radical surgery, including non-R0 resection due to the presence of very large tumors. Exclusion criteria were: receipt of preoperative chemotherapy, single postoperative 5-FU/capecitabine chemotherapy regimens or other regimens that contained neither 5-FU/capecitabine nor oxaliplatin; receipt of perioperative radiotherapy; and death within a 3-month period postoperatively.

The study was approved by the Ethics Committee of the Second Affiliated Hospital, Zhejiang University School of Medicine. Written informed consent was not obtained by patients for their clinical records to be used in this study. However, the patients' information was made anonymous prior to analysis.

### Statistical analysis

The following data were extracted: patients' demographic and cancer characteristics, surgical and perioperative complications, perioperative treatments, recurrences, and survival. The 7th edition of the TNM (tumor/node/metastasis) staging system was used. Data were analyzed with SPSS version 19.0. Survival was calculated from the date of operation. Gaussian distribution data were described by 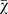 ± s and analyzed by *t* tests. Numeration data were analyzed by a χ^2^ test or Fisher's exact probability test. Local recurrence-free survival (RFS) was defined as the time to local pelvic recurrences, disease-specific survival (DSS) as the time to death caused by rectal cancer, and overall survival (OS) as the time to death. Kaplan-Meier censored survival curves were used to present survival data with log-rank *P* values. A two-sided *P* value of 0.05 or less was considered to indicate statistical significance.

## Results

### Baseline characteristics

On the basis of the inclusion criteria, the number of patients eligible for analysis was 285. However, 49 patients were excluded as 6 had received preoperative chemotherapy, 20 had received single 5-FU/capecitabine postoperative chemotherapy regimens, 4 had received other chemotherapy regimens, and 19 had received radiotherapy preoperatively or postoperatively. Thus, the analysis was performed on 236 eligible patients (Group S 135; Group SO 101), 10 of whom were subsequently lost to follow-up. All of the patients in Group SO received chemotherapy, including not only oxaliplatin but also 5-FU/capecitabine. The median follow-up time was 53.5 months (range, 3 to 124 months).

Patients in Group S were older, had a higher proportion with stage II disease, and had more perioperative complications than patients in Group SO (*P*<0.05). However, other characteristics such as gender, distance from the anal verge, surgical method, and pathologic type were balanced between the 2 groups (Table 1). Three patients received R2 resection due to the presence of very large tumors. All of the longitudinal resection margins were pathologically clear. None of the patients' circumferential resection margins (CRMs) were reported.

**Table 1:** Baseline demographic and cancer characteristics of the 236 patients who underwent radical surgery.

Characteristics	Group S (n = 135)	Group SO (n = 101)	*P* value
Gender, n (%):			0.131
Male	93 (68.89%)	60 (59.41%)	
Female	42 (31.11%)	41 (40.59%)	
Age, years (mean ± SD)	64.04±11.37	53.98±11.12	<0.001*
Distance to anal verge, cm (mean ± SD)	7.57±3.10	7.31±2.94	0.513
Surgical method, n (%):			0.944
AR	99 (73.88%)	76 (75.25%)	
APR	34 (25.37%)	24 (23.76%)	
Hartmann	1 (0.75%)	1 (0.99%)	
R0 resection, n (%):			1.000
Yes	133 (98.52%)	100 (99.01%)	
No	2 (1.48%)	1 (0.99%)	
Complications, n (%)			0.046*
Yes	15 (11.11%)	4 (3.96%)	
No	120 (88.89%)	97 (96.04%)	
Pathologic type, n (%):			0.380
Adenocarcinoma	127 (94.07%)	92 (91.09)	
Mucinous adenocarcinoma	8 (5.93%)	9 (8.91%)	
Differentiation, n (%):			0.074
Well-differentiated	64 (52.46%)	33 (35.87%)	
Moderately-differentiated	52 (42.62%)	52 (56.52%)	
Poorly-differentiated	6 (4.92%)	7 (7.61%)	
Lymph nodes (mean ± SD)	13.68±5.74	13.99±7.31	0.717
T stage, n (%):			0.336
1	0 (0%)	1 (0.99%)	
2	6 (4.44%)	9 (8.91%)	
3	66 (48.89%)	48 (47.52%)	
4	63 (46.67%)	43 (42.57%)	
N stage, n (%):			<0.001*
0	70 (51.85%)	27 (27.00%)	
1	44 (32.59%)	32 (32.00%)	
2	21 (15.56%)	41 (41.00%)	
TNM stage, n (%):			<0.001*
II	70 (51.85%)	27 (27.00%)	
III	65 (48.15%)	73 (73.00%)	

* Statistically significant difference between patient groups (*P*≤0.05).

AR, anterior resection; APR, abdominoperineal resection; Group S received surgery alone; Group SO received surgery and oxaliplatin-containing adjuvant chemotherapy.

### Effect of oxaliplatin-containing adjuvant chemotherapy on 5-year survival

**Figure 1:**
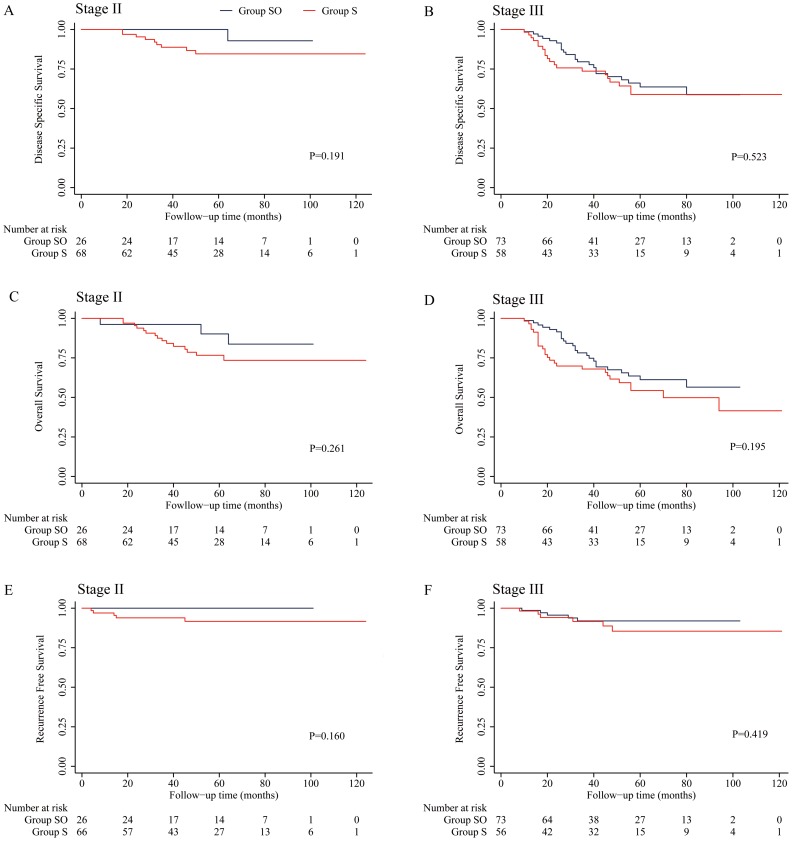
Kaplan-Meier curves for patients who received oxaliplatin-based adjuvant chemotherapy or surgery alone. A, C and E: patients with stage II disease. B, D and F: patients with stage III disease.

**Figure 2:**
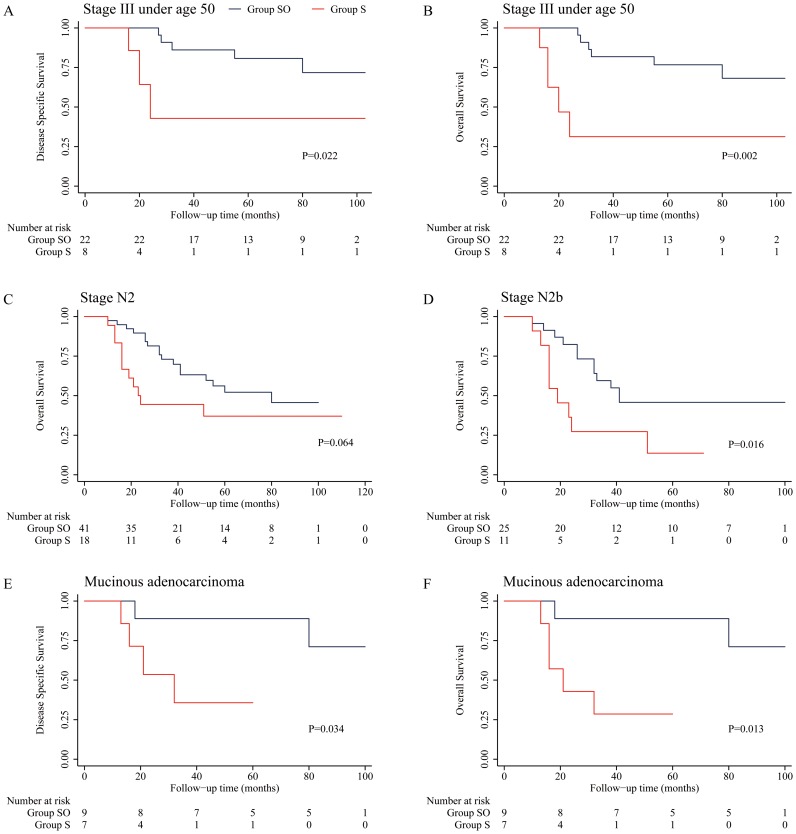
Kaplan-Meier curves for subgroups of patients who received oxaliplatin-based adjuvant chemotherapy or surgery alone. A and B: stage III patients under 50 years of age. C: patients with stage N2 disease. D: patients with stage N2b disease. E and F: patients with mucinous adenocarcinoma.

There were trends for the 5-year DSS and OS to be higher in patients with stage II and III disease who received oxaliplatin-containing adjuvant chemotherapy (Group OS) than in patients who received surgery only (Group OS) [Table 2], but the differences between the groups were not statistically significant (*P*>0.05). Subgroup analysis showed that both the DSS and OS of patients with stage III disease under 50 years age were higher in Group SO than in patients in Group S (*P*<0.05). Stratified analysis according to the N stage revealed a trend for patients with stage N2 disease in Group SO to have a better OS than those in Group S (52.2% vs 37.0%; *P* = 0.064). Further stratified analysis showed that the OS of patients with stage N2b disease in Group SO was increased in comparison with that of patients in Group S (45.8% vs 13.6%; *P* = 0.016). In addition, both the DSS and OS of patients with mucinous adenocarcinoma in Group SO were increased in comparison with patients in Group S (*P*<0.05) [Table 2; Figures 1 and 2].

**Table 2:** Five-year survival of the patients after rectal cancer resection.

Characteristics	5-Year DSS			5-Year OS		
	Group S n (%)	Group SO n (%)	Log-rank *P* value	Group S n (%)	Group SO n (%)	Log-rank *P* value
TNM stage:						
II	68 (84.6%)	26 (100%)	0.191	68 (76.6%)	26 (90.1%)	0.261
III	58 (58.9%)	73 (63.7%)	0.523	58 (54.4%)	73 (61.2%)	0.195
Age:						
<50 years	14 (73.8%)	31 (84.9%)	0.373	14 (62.9%)	31 (79.0%)	0.205
Stage II	6 (100%)	9 (100%)	NA	6 (100%)	9 (88.9%)	0.414
Stage III	8 (42.9%)	22 (80.7%)	0.022*	8 (31.3%)	22 (76.7%)	0.002*
≥50 years	112 (72.8%)	69 (66.6%)	0.449	112 (67%)	69 (62.9%)	0.947
Stage II	62 (82.9%)	17 (100%)	0.299	62 (74.2%)	17 (90.9%)	0.240
Stage III	50 (61.0%)	51 (54.0%)	0.705	50 (58.2%)	51 (52.2%)	0.994
T stage:						
2	6 (83.3%)	9 (100%)	0.317	6 (83.3%)	9 (100%)	0.317
3	64 (72.5%)	48 (70.5%)	0.760	64 (67.6%)	48 (64.0%)	0.627
4	56 (72.2%)	42 (70.1%)	0.388	56 (63.1%)	42 (68.4%)	0.974
N stage:						
N1	40 (64.3%)	32 (78.8%)	0.280	40 (62.5%)	32 (72.4%)	0.314
N1a	16 (67.5%)	16 (85.9%)	0.313	16 (67.5%)	16 (85.9%)	0.146
N1b+c	24 (61.9%)	16 (71.1%)	0.704	24 (59.2%)	16 (59.1%)	0.932
N2	18 (46.1%)	41 (52.2%)	0.333	18 (37.0%)	41 (52.2%)	0.064
N2a	7 (71.4%)	16 (52.5%)	0.609	7 (71.4%)	16 (52.5%)	0.609
N2b	11 (20.2%)	25 (45.8%)	0.161	11 (13.6%)	25 (45.8%)	0.016*
Pathologic type:						
Adenocarcinoma	119 (75.0%)	91 (70.9%)	0.578	119 (68.8%)	91 (66.4%)	0.930
Well-differentiated	60 (72.4%)	32 (70.8%)	0.830	60 (67.9%)	32 (64.2%)	0.650
Moderately- differentiated	49 (79.2%)	52 (75.8%)	0.765	49 (70.7%)	52 (72.4%)	0.902
Poorly-differentiated	5 (66.7%)	7 (42.9%)	0.551	5 (53.3%)	7 (42.9%)	0.949
Mucinous adenocarcinoma	7 (35.7%)	9 (88.9%)	0.034*	7 (28.6%)	9 (88.9%)	0.013*

* Statistically significant difference between patient groups (*P*≤0.05).

DSS, disease-specific survival; Group S received surgery alone; Group SO received surgery and oxaliplatin-containing adjuvant chemotherapy; NA, not available; OS, overall survival.

Further analysis according to the duration of adjuvant chemotherapy revealed that patients with stage N1a disease who received more than 8 weeks of chemotherapy in Group SO had a better DSS (100% vs 75.0%; *P* = 0.079) and OS (100% vs 68.8%; *P* = 0.032) than patients in Group S. In addition, the OS of patients with stage N2b disease who received more than 8 weeks of chemotherapy was improved in comparison with patients in Group S (44.4% vs 18.2%, *P* = 0.033).

Although the RFS of patients with stage II disease in Group SO was higher than in Group S, the difference was not statistically significant; the 5-year local recurrence rates in the 2 groups were 0% and 8.3%, respectively (*P* = 0.160). Similarly, there was no difference in RFS between the 2 groups for patients with stage III disease; the 5-year local recurrence rates for these patients were 8.1% and 14.6%, respectively (*P* = 0.419) [Table 3]. No subgroups of patients who achieved a lower local recurrence rate following receipt of oxaliplatin-containing adjuvant chemotherapy were identified (Figure 1 and Table 3).

**Table 3:** Five-year local recurrence-free survival of the patients after rectal cancer resection.

Characteristics	5-Year RFS		
	Group S n (%)	Group SO n (%)	Log-rank *P* value
TNM stage:			
II	66 (91.7%)	26 (100%)	0.160
III	56 (85.4%)	73 (91.9%)	0.419
Age:			
<50 years	13 (100%)	31 (95.8%)	0.564
Stage II	6 (100%)	9 (100%)	NA
Stage III	7 (100%)	22 (94.4%)	0.739
≥50 years	109 (88.0%)	69 (93.5%)	0.369
Stage II	60 (90.8%)	17 (100%)	0.225
Stage III	49 (84.4%)	51 (90.9%)	0.567
T stage:			
2	6 (100%)	9 (100%)	NA
3	64 (86.7%)	48 (90.0%)	0.649
4	52 (90.4%)	42 (97.5%)	0.268
N stage:			
N1	40 (83.8%)	32 (96.7%)	0.166
N1a	16 (100%)	16 (100%)	NA
N1b+c	24 (73.3%)	16 (92.9%)	0.321
N2	18 (92.3%)	41 (87.8%)	0.800
N2a	7 (83.3%)	16 (85.9%)	0.799
N2b	11 (100%)	25 (89.7%)	0.448
Pathologic type:			
Adenocarcinoma	116 (89.6%)	91 (94.8%)	0.270
Well-differentiated	59 (90.1%)	32 (92.6%)	0.679
Moderately-differentiated	47 (86.8%)	52 (97.8%)	0.097
Poorly-differentiated	5 (100%)	7 (85.7%)	0.398
Mucinous adenocarcinoma	6 (80.0%)	9 (87.5%)	0.572

Group S received surgery alone; Group SO received surgery and oxaliplatin-containing adjuvant chemotherapy; NA, not available; RFS, local recurrence-free survival.

## Discussion

In the 1990s, a number of clinical trials demonstrated that postoperative radiotherapy reduced local recurrences in patients with locally-advanced rectal cancer [11], [12]. Since then, radiotherapy has become the cornerstone of adjuvant therapy for advanced rectal cancer in western countries. During the first decade of the 21st century, preoperative radiochemotherapy with 5-FU became the standard adjuvant therapy for locally-advanced and resectable rectal cancer due to its efficacy in reducing local recurrences and improving survival [4]–[6]. At the same time, chemotherapy containing oxaliplatin also became the standard adjuvant therapy for resectable advanced colon cancer [1]–[3]. Therefore, several clinical trials tried to improve the treatment effect of rectal cancer by adding oxaliplatin to preoperative radiochemotherapy. However, the results were disappointing because of both increased adverse events and no improvement in the tumor response [7], [8]. As a result, there has been no evidence to support the use oxaliplatin as adjuvant therapy for locally-advanced rectal cancer. Nevertheless, it has become evident that there are still several issues regarding preoperative radiotherapy that remain to be clarified. For example, patients who receive preoperative radiotherapy suffer from sexual dysfunction, proctitis, enteritis and cystitis, especially females with a low body mass index [13], [14]. Moreover, radiotherapy for patients with T3N0 rectal cancer has also been questioned [15], [16]. Whether intensive chemotherapy without radiotherapy can improve the prognosis of locally-advanced rectal cancer remains unknown.

In this retrospective study, we observed survival differences between patients with locally-advanced curable rectal cancer who received surgery alone and those who received surgery and oxaliplatin-containing adjuvant chemotherapy. Our findings showed that some patients with stage III rectal cancer can benefit from oxaliplatin-containing adjuvant chemotherapy, including: (1) patients with stage III disease less than 50 years of age; (2) patients with stage N2 disease, especially those with stage N2b disease; and (3) patients with stage N1a disease who received more than 8 weeks of chemotherapy. The DSS and OS of patients with stage II disease also showed a trend towards improvement, but the differences versus Group S did not reach statistical significance. It was of interest that no specific subgroup of patients achieved a reduced local recurrence rate with oxaliplatin-containing adjuvant chemotherapy. This indicates that the survival benefit that was achieved might be the result of reduced distant metastases rather than decreased local recurrences.

At present, there is no direct evidence to support the use of oxaliplatin-based chemotherapy alone for middle and low rectal cancers. The key role of radiotherapy has been well established, and all trials that have studied oxaliplatin in combination with radiotherapy have failed, which, prior to this study, has made it ethically difficult to assess the effect of single oxaliplatin-containing adjuvant chemotherapy regimens for rectal cancer in clinical trials. In comparison with the reported results with standard preoperative radiochemotherapy, the findings of this study indicate that single oxaliplatin-containing adjuvant chemotherapy provides non-inferior overall survival. The 5-year overall survival rate of patients in Group SO was 68.8% (90.1% for stage II disease and 61.2% for stage III disease). In the NSABP R-03 trial, the 5-year OS rate was 74.5% for patients who received preoperative radiochemotherapy that contained 5-FU [5]. In the EORTC 22921 trial, patients with locally-advanced resectable rectal cancer were randomly assigned to receive preoperative radiotherapy, preoperative chemoradiotherapy, preoperative radiotherapy and postoperative chemotherapy, or preoperative chemoradiotherapy and postoperative chemotherapy. The combined 5-year OS rate for all 4 groups was 65.2% [17]. Our results indicate that oxaliplatin-containing adjuvant chemotherapy may be as effective as the present standard of radiochemotherapy. In view of the non-inferior 5-year overall survival for patients in Group SO in this study (61.2% for patients with stage III disease and 76.7% for patients with stage III disease under 50 years of age), it seems reasonable to perform studies of oxaliplatin-containing chemotherapy instead of radiotherapy in some patients, e.g. young females because of concerns over ovarian dysfunction with radiotherapy, and patients with contraindications to radiotherapy [5], [6]. Moreover, patients with rectal cancers in which the surgical CRM is found to be clear by magnetic resonance imaging (MRI) might also be able to receive intensive adjuvant chemotherapy and thereby avoid radiotherapy [18]. The feasibility of this approach needs to be confirmed by prospective, randomized trials.

Recently, several studies have reported results with the application of oxaliplatin in rectal cancer instead of combining it with preoperative radiotherapy. The large multicenter, phase II ADORE study used postoperative oxaliplatin-based adjuvant chemotherapy (FOLFOX) in patients who had received preoperative radiochemotherapy [19]. The 3-year disease-free survival was 71.6% in patients who received FOLFOX postoperative adjuvant chemotherapy, as compared with 62.9% in patients who received 5-FU/leucovorin (*P* = 0.047). In a subgroup analysis, patients with yp stage III, ypN1b, ypN2, and minimally-regressed tumors benefited more from FOLFOX than 5-FU/leucovorin. Two other recent studies have explored the question of whether intensive chemotherapy containing oxaliplatin could replace preoperative radiotherapy. A small phase II study conducted in New York reported a pathologic complete response rate of 25% and a 4-year overall survival rate of 91% for patients with locally-advanced rectal cancer who received FOLFOX with bevacizumab as preoperative therapy without routine radiotherapy [20]. Another phase II study conducted in Japan reported that neoadjuvant therapy using mFOLFOX6 instead of radiotherapy was a safe and efficacious treatment option for rectal cancer, with a pathologic complete response rate of 10.3% and an R0 resection rate of 91.0% [21]. All of these findings indicate that oxaliplatin-containing adjuvant chemotherapy may be effective in rectal cancer when synchronous use of preoperative radiotherapy is to be avoided.

This study had several limitations. Firstly, as it was a retrospective study with only a small sample, there was selection bias in the patients included. As described above, patients in Group S were older, contained a higher proportion with stage II disease, and had more perioperative complications than patients in Group SO (*P*<0.05). Therefore, patients who were young with a good physical status and a higher risk of recurrences were more liable to receive adjuvant chemotherapy. Consequently, to eliminate the effects of selection bias, we used a stratification analysis.

Secondly, no CRM status was reported. As we reported in 2009 [10], this is a serious shortcoming of the treatment of rectal cancer in China, even in university-affiliated hospitals. However, for patients in Group S, the high number of lymph nodes harvested (mean 13.68), the non-inferior 5-year local recurrence rate (8.3% in stage II disease and 14.6% in stage III disease), and the disease-specific survival rate (84.6% in stage II disease and 58.9% in stage III disease) were comparable to the results reported by Heald et al. [22], indicating the good quality of surgery at our center. Thirdly, it was not feasible to compare outcome differences between patients who received oxaliplatin-containing adjuvant chemotherapy and those who received perioperative radiotherapy in this study, as only 19 patients who had very large tumors or obvious lymph node metastases received radiotherapy.

In conclusion, we found that adjuvant chemotherapy containing oxaliplatin without radiotherapy can improve the survival of patients with locally-advanced low and middle rectal cancers in comparison with observation. Randomized, prospective trials are warranted to confirm this benefit of oxaliplatin for rectal cancer.
